# Work-related social media exposure and teacher burnout in pre-pandemic China: the mediating role of work-to-family conflict

**DOI:** 10.3389/fpubh.2025.1490810

**Published:** 2025-06-11

**Authors:** Aonan Wu, Qianqian Mao, Dong AN

**Affiliations:** ^1^College of Cultural Communication, Shandong Youth University of Political Science, Jinan, China; ^2^School of Event and Economic Management, Shanghai Institute of Tourism, Shanghai, China; ^3^School of Arts, Southeast University, Nanjing, China

**Keywords:** burnout, Chinese middle school teacher, social media exposure, work-to-family conflict, COVID-19

## Abstract

Teacher burnout negatively impacts educational quality and the interpersonal relationships among teachers, parents, and students. In the context of increasing integration of social media in education, this brief report examines the relationship between work-related social media exposure (SME) and burnout. Adopting a cross-sectional survey design, data were collected via an online questionnaire in early 2019 from middle school teachers (*N* = 141) in China’s Yangtze River Delta Economic Zone. Statistical analyses were performed using SPSS 26.0 and its PROCESS macro for mediation analysis. The results reveal significant correlations between work-related SME and burnout, with work-to-family conflict serving as a partial mediator. Drawing on the Conversation of Resources (COR) theory, this study highlights the detrimental effects of excessive work-related SME on teachers’ mental health and professional functioning, evening before the onset of the COVID-19 pandemic. These findings underscore the urgency for educational policymakers to develop targeted interventions, including (1) institutional protocols to mitigate SME-induced boundary permeability, and (2) evidence-based strategies for preserving work-family boundary.

## 1 Introduction

Over the past decade, social media has become deeply embedded within individuals’ daily routines, significantly influencing informal interactions, institutional structures, and professional practices (1–4). The pervasive integration of digital technologies into work environments has created new forms of technostress—a phenomenon in Which ICT demands exceed users’ adaptive capacities, resulting in psychological strain (5–7). This phenomenon has been further exacerbated since the onset of the COVID-19 pandemic in early (8), as the combined forces of physical isolation and digital immediacy have intensified workplace reliance on social media (9–12). Notably, the education sector has experienced unprecedented technologization of pedagogical practices during this period (13–15).

While pandemic-era studies highlight the psychological toll of excessive digital engagement among employees (16, 17), they often create the illusion that crises stemming from digital communication and online remote work may diminish as the COVID-19 stabilizes (18, 19). However, a critical research gap persists: the lack of comparative analyses between pre-pandemic and pandemic educational contexts. Such comparisons could help isolate COVID-19-specific variables and systematically examine the operational mechanisms of technological invasion (e.g., social media exposure) within pedagogical ecosystems. Even before COVID-19, researchers identified chronic technology invasion as a key driver of work-family boundary dissolution and emotional exhaustion (20). While social media may be perceived as a resource for building social networks (21), it can also function as a latent resource drain, akin to other emergent technologies (22). The Conservation of Resources (COR) theory, proposed by Hobfoll (23), posits that resources are the key determinant of whether events or stimuli are perceived as stressful, and their availability directly influences individuals’ coping strategies in stressful situations. Unlike the Job Demands-Resources (JD-R) model, COR emphasizes not only workplace resources but also the role of personal resources in employee adaptation. Core tenets of COR theory include: (1) individuals strive to protect existing resources; (2) they actively accumulate resources to manage high-demand situations; (3) the loss or threatened loss of these resources may induce stress (24). Resources are categories into four distinct types: objects, conditions, personal characteristics, and energy (23).

According to the COR theory, burnout emerges through a sustained process of continuous resource loss. Social media usage in professional contexts is increasingly conceptualized as a “resource” rather than “demands” (25), where such social media resources leading to the blurring of work-family boundaries, thereby contributing to burnout. This study applies the Conservation of Resources (COR) theory to explore teachers’ burnout and social media exposure among teachers in the pre-pandemic context, incorporating the depletion of personal social media resources as a predictor of burnout. Notably, Chinese middle school teachers—predominantly “digital natives” (45) —confront unique pressures stemming from near-constant parent-teacher communication through platforms such as WeChat and QQ (8). This indicates that middle school teachers in China experience exceptionally severe technological intrusion and resource loss resulting from social media exposure. Therefore, we employ work-to-family conflict as a mediator to examine the SME-burnout relationship.

In this study, we conceptualize social media as a dual-nature resource—a novel technological application in workplace contexts—that may simultaneously trigger technological boundary invasion into teachers’ familial domains and accelerate personal resource depletion. While prior research has established that work-related information technology (IT) use during non-work hours induces individual resource attrition through techno-invasion mechanisms, the extent to which work-related social media exposure operates as a latent techno-stressor—potentially intensifying job burnout and eroding work-family demarcations among Chinese middle school teachers—remains empirically underdeveloped. Using a cross-sectional survey methodology conducted in middle school within the Yangtze River Delta Economic Zone in China, this study theoretically explains the impact of social media as a form of techno-invasion and personal resource on Chinese middle school teachers’ burnout prior to the COVID-19 pandemic, emphasizing the blurred boundaries between work and family in shaping this relationship. Our findings reveal the following:

1. Drawn upon COR theory, our study extends previous research on burnout and the use of social media technologies among teachers in the pre-pandemic context. It identifies a significant correlation between work-related SME overload and burnout among middle school teachers.
2. This analysis demonstrates that work-to-family conflict partially mediates the relationship between work-related SME and burnout, particularly in the dimensions of emotional exhaustion and professional efficacy. It underscores the critical influence of work-to-family conflict as a key driver of occupational stress.
3. The study systematically excluded acute public health disruptions to reveal significant associations between burnout and “digital work boundary permeability.” These findings engage with post-pandemic scholarship that predominantly attributes comparable outcomes to remote work proliferation. By differentiating institutional stressors from situational stressors, the analysis demonstrates that chronic professional attrition originates primarily from endemic technological saturation rather than transient crisis-driven usage patterns.

Moreover, the extensive use of digital communication and social media in the education sector has been shown to blur boundaries between teachers’ professional and personal lives, potentially triggering occupational crises. These findings serve as a critical reminder that administrators must actively monitor and address the impact of work-related SME and other forms on teachers’ job performance and mental well-being, even as the COVID-19 pandemic subsides.

## 2 Method

A sample of participants was recruited from middle schools in the Yangtze River Delta Economic Zone between March 15 and March 25, 2019. Data were collected via survey method through the online platform *Questionnaire Star* (*wenjuan xing*) to test the research model. To be eligible, participants were required to have prior experience with work-related social media engagement (SME), such as participating in parents’ groups or similar professional activities. Ethical procedures were strictly followed, ensuring participant anonymity as well as the right to voluntary and informed participation. Demographic and work-related information—including teaching experience, education level, marital status, and monthly income—was also collected.

One of the main challenges among contemporary teachers in balancing time and resources between paid work and family responsibilities. This phenomenon has been extensively examined in work–family literature through the lens of work–family conflict (26). As noted in some prior studies, work–family conflict manifests bidirectional interference: work negatively impacting family domains (work-to-family conflict) and family demands adversely affecting work performance (family-to-work conflict) (27–29). Since the present study focuses mainly on work stressors, only work-to-family conflict was evaluated. Work-to-family conflict was measured using a five-item scale adapted from Netemeyer et al. (30), with higher scores indicating greater levels of conflict. All items were rated on a 5-point Likert scale ranging from 1 (“never” or “strongly disagree”) to 5 (“always” or “strongly agree”).

Burnout was measured using the Maslach Burnout Inventory (31), comprising 15 items across three dimensions: emotional exhaustion, cynicism, and professional efficacy. Similar to the work-to-family conflict scale, all items were scored on a 5-point Likert scale from 1 (“never” or “strongly disagree”) to 5 (“always” or “strongly agree”). Higher scores in emotional exhaustion and cynicism dimensions indicated greater levels of burnout, whereas lower scores in professional efficacy also reflected higher burnout levels. Work-related social media exposure (SME) was evaluated using three items: (1) “To what extent do you use social media to address work-related issues (e.g., communicating with students’ parents)?” (2) “How frequently do you use social media to handle work-related matters?” and (3) “How much time do you spend on work-related social media messages every day?” These items were scored on a 5-point Likert scale from 1 (“never”) to 5 (“always”), with higher scores indicating greater levels of work-related social media exposure.

## 3 Results

All statistical analyses were conducted using SPSS software (v26.0), with a two-tailed approach applied to all statistical tests. Before the regression analysis, we confirmed that the data satisfied the basic assumptions of regression: linearity, homogeneity of variation, and normality of multicollinearity. After regression analysis, a Sobel test using PROCESS macro in SPSS was performed to verify the significance of mediating effect sizes. A total of 157 participants were initially recruited for the study. After excluding outliers, the final sample comprised 141 participants (37 males and 104 females) who were included in subsequent data analysis. Table 1 provides an overview of the demographic and occupational characteristics of the participants, including educational attainment (categorized as junior college, graduate, or postgraduate), marital status (classified as single, separated/married, or cohabiting), and gender (male/female). Additionally, Table 1 displays the distribution of burnout dimensions, including burnout dimensions—emotional exhaustion, cynicism, and professional efficacy—across the demographic and occupational categories.

**Table 1:** Demographic and characteristics of participants, and distribution of burnout dimensions.

Variable	*N* (%)	Emotional exhaustion M (SD)	Cynicism M (SD)	Professional Efficacy M (SD)
Education				
Graduate	101 (71.63)	13.12 (5.17)	10.04 (3.37)	15.16 (3.96)
Postgraduate	40 (28.37)	15.65 (6.16)	10.10 (3.56)	15.05 (3.61)
Marital status				
Single/Separated	46 (32.6)	13.28 (5.45)	10.07 (3.43)	14.28 (3.67)
Married/Cohabitation	95 (67.4)	14.10 (5.63)	10.05 (3.42)	15.53 (3.89)
Monthly income				
<3,000	54 (38.30)	13.11 (5.78)	9.46 (3.28)	14.96 (3.32)
3,000–5,000	56 (39.72)	14.23 (4.98)	10.80 (4.18)	15.49 (4.18)
>5,000	31 (21.2)	14.07 (5.36)	9.30 (2.88)	14.81 (3.78)

*N* = sample size, M = mean, SD = standard deviation.

Statistical analyses were conducted using a three-step approach to examine the relationships among work-related social media exposure (SME), work-to-family conflict, and the three dimensions of burnout. Initially, bivariate relationships between these variables were assessed through Pearson correlation analysis. Subsequently, hierarchical regression analyses were performed to investigate the predictive effects of work-related SME and work-to-family conflict on the three dimensions of burnout. The hierarchical regression model was constructed in three sequential steps: In Step 1, control variables such as education level, marital status, and income were included in the model to account for potential confounding effects. In Step 2, the independent variable, work-related SME, was entered to assess its direct impact. In Step 3, work-to-family conflict was added to the model to evaluate its additional explanatory power. Furthermore, the Sobel test was used to verify the mediating effect.

### 3.1 Correlations among work-related SME, work-to-family conflict and burnout

The results of the Pearson correlation analysis are summarized in Table 2. Work-related SME demonstrated significant correlations with all dimensions of burnout. Specifically, SME was positively associated with both emotional exhaustion and cynicism, while it was negatively correlated with professional efficacy. In addition, work-related SME showed a positive correlation with work-to-family conflict.

**Table 2:** Correlation between SME, burnout, and work-to-family conflict.

Variables	Mean	SD	α	1	2	3	4	5
Work-related SME	3.14	1.14	-	1				
Emotioanl Exhaustion	13.84	5.58	0.90	0.34**	1			
Cynicism	10.06	3.41	0.79	0.18*	0.63**	1		
Professional Efficacy	15.13	3.86	0.82	−0.25**	0.19*	0.41**	1	
Work-to-family Conflict	13.25	5.54	0.96	0.26**	0.60**	0.65**	0.21*	1

M = mean, SD = Standard Deviation, α = Cronbach’s alpha coefficient. **. *p* < 0.01 (two-tailed), *. *p* < 0.05 (two-tailed).

### 3.2 The mediating role of work-to-family conflict in the relationship between SME and burnout

The results of the hierarchical linear regression analysis are detailed in this section, with Table 3 presenting the findings. The analysis examines the predictors of burnout, including demographic variables, work-to-family conflict, and work-related SME, across its three dimensions: emotional exhaustion, cynicism, and professional efficacy.

**Table 3:** Regression analysis predicting burnout from work-related SME and work-to-family conflict.

Variables	Emotional Exhaustion			Cynicism			Professional Efficacy		
	β	F	*p*	β	F	*p*	β	F	*p*
Education	0.21**	2.38	0.002	0.03	0.10	0.64	−0.01	1.15	0.93
Marital Status	0.09		0.17	0.04		0.57	0.17*		0.03
Income	−0.06		0.38	−0.08		0.27	0.40		0.63
Work-related SME	0.18*	5.14	0.027	0.001	1.11	0.99	−0.28**	2.50	0.004
Work-to-Family Conflict	0.56**	17.92	0.000	0.66**	16.70	0.000	0.31***	4.58	0.000
R^2^	0.45			0.43			0.17		
Δ R^2^	0.42			0.40			0.13		

β = regression coefficient, F = F-statistic, *p* = *p*-value, R^2^ = Coefficient of Determination, Δ R^2^ = Change in R-squared. **. *p* < 0.01 (two-tailed), *. *p* < 0.05 (two-tailed).

In predicting emotional exhaustion, education level was found to have a significant relationship with emotional exhaustion. Work-related SME was positively associated with emotional exhaustion, while work-to-family conflict, introduced in a subsequent step, also showed a significant effect. The overall model was statistically [*F* (6, 134) = 17.92, *p* < 0.001] and explaining 42% of the variance in emotional exhaustion. Using the same analytical approach, the analysis for cynicism, as shown in Table 3, indicated that work-related SME was not significantly associated with cynicism, and the model was not statistically significant. In predicting professional efficacy, marital status emerged as a significant predictor. Work-related SME negatively predicted professional efficacy, and work-to-family conflict, added in a later step, also showed a significant effect. The overall model was statistically significant [*F* (6, 134) = 4.58, *p* < 0.001], accounting for 13% of the variance in professional efficacy.

## 4 Discussion

Since the outbreak of the Covid-19 pandemic, many frontline teachers have encountered challenges such as emotional exhaustion, high task pressure, and role ambiguity (32). These challenges are closely related to digital communication, promoting extensive discussions on the role of digital competencies and new technologies in teacher burnout during the pandemic context (33). However, ss the Covid-19 crisis begins to stabilize, it remains uncertain whether the effects of digital communication, including social media exposure (SME), have dissipated or diminished. Research by Fang et al. (34) indicates that using social media for work outside of working hours significantly contributes to teacher burnout. Interestingly, our study reveals that the association between SME and burnout predates the pandemic. Data from 2019 show that work-related social media exposure (SME) was already significantly correlated with burnout among middle school teachers, broadening existing understandings of SME as a risk factor for occupational stress. This finding challenges the widespread narrative that digital burnout may emerge primarily as a pandemic-specific crisis. By identifying SME as a persistent precursor to burnout, independent of pandemic conditions, our research reframes the discussion of occupational stress in the digital age.

Prior literature has primarily focused on work-to-family conflict as a mediating factor in the relationship between various influences—such as job stress, organizational climate, psychological capital, and cognitive appraisal—and burnout (35–38). Consistent with this, our findings indicate that work-to-family conflict partially mediates the relationship between work-related SME and burnout, particularly in the dimensions of emotional exhaustion and professional efficacy. This further highlights the importance of work–family conflict as a significant factor influencing occupational stress. Moreover, the extensive use of digital communication and social media within the education sector can easily blur the boundaries between teachers’ professional and personal lives, potentially leading to the risk of occupational crises. Notably, our data reveal significant correlations between teachers’ educational backgrounds, marital status, and the prevalence of burnout. This finding emphasizes the importance for administrators to fully consider personal circumstances of teachers in order to mitigate the risk of burnout crises.

According to Conservation of Resources (COR) theory, individuals strive to acquire, protect, and maintain resources to cope with stress, while resource loss (or threats to resources) serves as a core mechanism driving burnout. Building on our findings, we observe that work-related social media exposure (SME) compels teachers to continuously invest time and cognitive resources (e.g., responding to parent messages around the clock or managing workgroup tasks). This “technological intrusion” blurs the boundaries between work and family domains, creating resource strain (39). As COR theory suggests, when resource investments (e.g., social media interactions during non-work hours) are not be replenished through restorative activities (e.g., quality family time), SME triggers a resource imbalance, ultimately leading to emotional exhaustion and reduced professional efficacy.

Work–Family Conflict reflects the competitive allocation of resources between work and family roles. When teachers handle work-related social media tasks during family time, they not only deplete resources originally allocated to familial responsibilities but also experience a failure in resource protection (40). These persistent cross-boundary demands may trap middle school teachers in a cycle of “resource loss spirals” (41), where ongoing resource depletion exacerbates stress and burnout. Importantly, our study reveals that the association between SME and burnout significantly predated the pandemic. This finding suggests that the pandemic did not introduce novel stressors but rather accelerated pre-existing resource depletion processes through enforced digitization (e.g., remote work and online teaching). COR theory emphasizes the cumulative effects of resource erosion, highlighting that even low-intensity but chronic resource depletion can gradually undermine teachers’ psychological resilience over time (39). While the pandemic may have amplified these challenges, it also normalized them, making such issues appear less visible or urgent. This normalization risks overshadowing the ongoing crises and demands faced by teachers, which existed long before the pandemic and continue to persist within the broader narrative of occupational stress in the teaching profession.

Based on the results, we can adopt strategies from two approaches—resource conservation and resource replenishment—to alleviate teachers’ social media exposure stress. First, educational institutions should prioritize digital boundary training to equip teachers with empirically supported strategies for mitigating digital intrusion, such as scheduled notification silences during family time or platform-specific availability markers. Research by Fast (42) demonstrated that digital disconnection practices reduced cognitive overload in remote workers, suggesting their applicability to educational contexts. Second, systemic reforms must institutionalize safeguards against perpetual connectivity. Drawing lessons from Enli and Fast (43) which legally protects people’s right to disconnect after digital invasion, one approach could involve establishing standardized response windows for non-urgent communications. For instance, schools could implement policies requiring teachers to respond to parent inquiries only within a designated 6-h timeframe, thereby reducing the burden of constant availability. Third, tailored support systems are necessary to address the heightened burnout vulnerability observed in specific subgroups of teachers. For novice educators, mentorship programs that pair them with experienced teachers skilled in navigating digital boundaries could help mitigate transitional stressors. Additionally, districts could introduce credential recognition programs that translate digital engagement metrics (e.g., discussion forum facilitation) into professional development credits. This would align teachers’ online labor with career advancement opportunities, offering tangible rewards for their efforts. Such targeted approaches not only acknowledge the heterogeneity of teachers’ digital experiences but also address systemic inequities in burnout vulnerability. Moreover, educational administrators should actively regulate teachers’ exposure to work-related social media by fostering a shared “consensus” among teachers, parents, and students regarding the use of these platforms. Administrators must recognize the emotional investment teachers make in maintaining their professional identity on social media and strive to eliminate the “always online” phenomenon. Providing teachers with greater personal space and clearly defining the boundaries between work and family life represent effective strategies for reducing occupational burnout.

The current study, while offering novel insights into pre-pandemic patterns of teacher burnout, is subject to several methodological limitations that warrant careful consideration. First, the cross-sectional design, with its temporal specificity, limits causal interpretations. By relying solely on 2019 data, the study cannot capture the pandemic-induced accelerations of digital adoption pattern. Second, the participating teachers are relatively young in terms of teaching experience, and the differences in social media exposure among more experienced teachers were not examined. Third, the study was conducted in an economically developed region with relatively high levels of social media literacy, raising questions about the generalizability of findings to teachers in more remote or underdeveloped areas. Additionally, the disproportionately high representation of female participants may skew findings related to gender influences on burnout (44). Lastly, the reliance on self-reported data for measuring work–family conflict (WFC) introduces potential measurement bias. Without incorporating perspectives from spouses or partners, the study risks inflating common-method variance and overlooks critical aspects of boundary violation dynamics, such as how family members perceive and adapt to teachers’ digital intrusions.

To address these limitations and advance the field, three interconnected research priorities emerge. First, future studies should adopt longitudinal mixed-methods designs to disentangle the evolution of digital stressors across distinct pandemic phases. For instance, combining experience sampling methodology (ESM) to track real-time SME exposures during acute pandemic periods (2020-2022) with archival analyses of pre-pandemic policy documents (2015–2019) could map the institutional normalization of constant connectivity. Second, geospatial comparisons should investigate how regional digital divides moderate the relationship between SME and burnout. Multilevel modeling could test whether teachers in broadband-deficient rural areas experience heightened anticipatory stress due to intermittent connectivity, thus providing a deeper understanding of how infrastructural disparities shape digital stress. Third, technological intervention studies should empirically evaluate AI-mediated solutions to digital overload. Such interventions could provide actionable strategies to mitigate the physiological and psychological impacts of digital stress.

## 5 Conclusion

Our brief report highlights the relationship between teachers’ work-related social media exposure (SME) and burnout prior to the COVID-19 pandemic. The data reveal a significant correlation between work-related SME and burnout among Chinese middle school teachers, with work-to-family conflict partially mediating the relationship. Three pivotal insights emerge from our findings: First, the overload of work-related SME among Chinese middle school teachers in 2019 demonstrated a strong correlation with burnout severity, particularly in dimensions of emotional exhaustion and diminished professional efficacy. Second, work-to-family conflict emerged as a partial mediator in this relationship, suggesting that blurred digital boundaries systematically erode teachers’ psychological detachment capacity—a mechanism exacerbated by institutional norms of constant connectivity. Third, our findings indicate that even before the pandemic, the overload of work-related SME had already significantly affected teachers’ mental health and professional functioning, underscoring the pre-existing vulnerabilities amplified by the pandemic. The study systematically excluded acute public health disruptions to reveal significant associations between burnout and “digital work boundary permeability.” These findings engage with post-pandemic scholarship that predominantly attributes comparable outcomes to remote work proliferation. By differentiating institutional stressors from situational stressors, the analysis demonstrates that chronic professional attrition originates primarily from endemic technological saturation rather than transient crisis-driven usage patterns.

This report enhances our understanding of how work-related digital communication influences burnout within the teaching community and highlights the necessity for administrators to address the professional crises stemming from excessive social media exposure. While limited by its pre-pandemic temporal scope and urban sample bias, this study provides a foundational framework for future investigations. Longitudinal studies tracking SME’s evolving impact across pandemic phases, cross-cultural comparisons of digital adaptation strategies, and controlled trials of AI-mediated communication tools represent critical for future research. Ultimately, this work underscores the need to reconceptualize digital connectivity in education—not as an unavoidable occupational hazard, but as a modifiable stressor that demands systemic, human-centered interventions. By addressing these challenges proactively, educational institutions can mitigate burnout risks while harnessing digital tools’ transformative potential responsibly.

## Data Availability

The original contributions presented in the study are included in the article/supplementary material, further inquiries can be directed to the corresponding author.
